# Supplementary figures and images for: Resection is responsible for loss of transcription around a double-strand break in Saccharomyces cerevisiae
Source: eLife. 2015 Jul 31;4:e08942. doi: 10.7554/eLife.08942 (PMC4541074; doi:10.7554/eLife.08942)

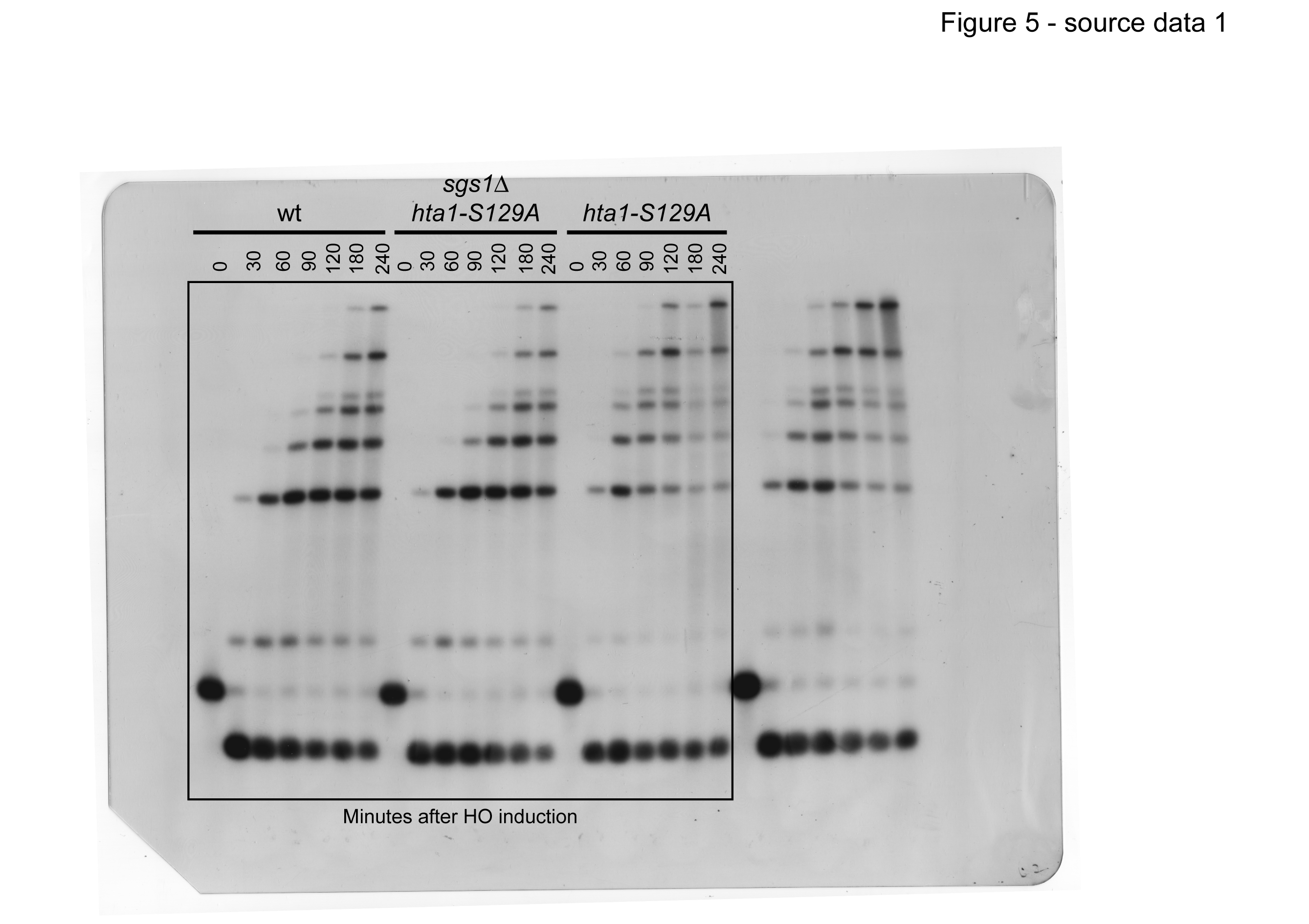

Supplement: Figure 5—source data 1. — DOI: http://dx.doi.org/10.7554/eLife.08942.009 [file elife08942s002.tif]

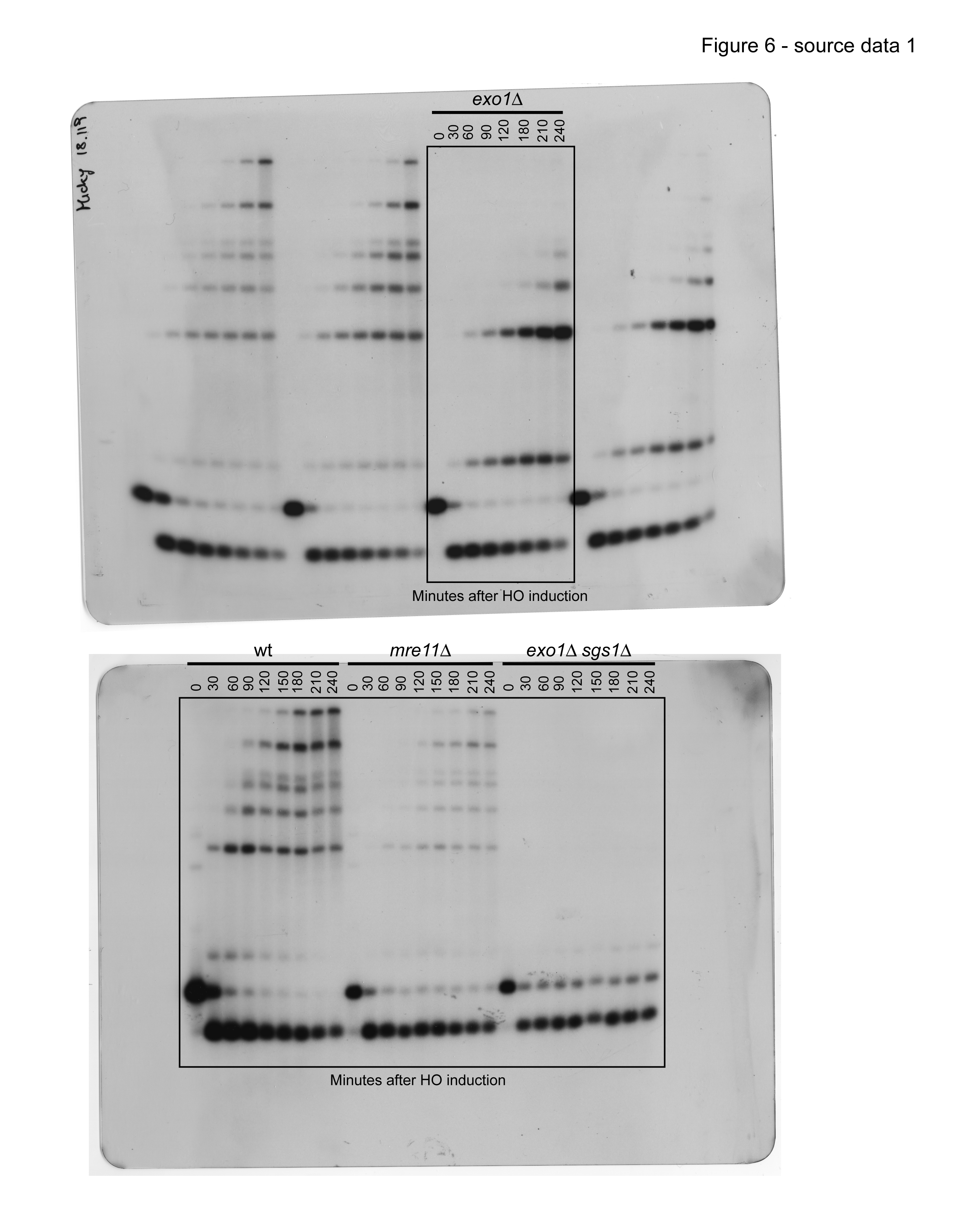

Supplement: Figure 6—source data 1. — DOI: http://dx.doi.org/10.7554/eLife.08942.011 [file elife08942s003.tif]
