# Supplementary material for: Resection is responsible for loss of transcription around a double-strand break in Saccharomyces cerevisiae
Source: eLife. 2015 Jul 31;4:e08942. doi: 10.7554/eLife.08942 (PMC4541074; doi:10.7554/eLife.08942)
Supplement: Supplementary file 1. — Saccharomyces cerevisiae strains used in this study. DOI: http://dx.doi.org/10.7554/eLife.08942.013 [file elife08942s004.doc]

**Supplementary file 1.** *Saccharomyces cerevisiae* strains used in this study.

| Strain | Relevant genotype | Source |
| --- | --- | --- |
| JKM139 | *MATa ho hml****∆****::ADE1 hmr****∆****::ADE1 ade3::GAL-HO ade1-100 leu2-3;112 lys5 trp1::hisG ura3-52* | Lee et al., 1998 |
| 184/10A | JKM139 *sml1∆::KANMX4 mec1∆::HIS3* | Mantiero et al., 2007 |
| YLL1794 | JKM139 *tel1∆::NATMX* | Clerici et al., 2014 |
| 185/15C | JKM139 *sml1∆::KANMX4 mec1∆::HIS3 tel1∆::NATMX* | This study |
| DMP5396/4C | JKM139 *hta2∆::NATMX* | Clerici et al., 2014 |
| DMP5129/19B | JKM139 *hta2∆::NATMX hta1-S129A::URA3* | Clerici et al., 2014 |
| YLL3405 | JKM139 *hta2∆::NATMX hta1-S129A::URA3 sgs1∆::NATMX* | This study |
| YLL1769 | JKM139 *mre11∆::NATMX* | Clerici et al., 2006 |
| YLL1540 | JKM139 *exo1∆::LEU2* | Clerici et al., 2006 |
| DMP5689/7C | JKM139 *rad9∆::KANMX4* | Clerici et al., 2014 |
| DMP5706/11D | JKM139 *exo1∆::LEU2 sgs1∆::NATMX* | Clerici et al., 2014 |
| YLL3542 | JKM139 *RPB2-3HA::TRP1* | This study |
| YLL3543 | JKM139 *RPB2-3HA::TRP1 exo1∆::LEU2 sgs1∆::NATMX* | This study |
| YFP17 | *mata****∆****::hisG ho hml****∆****::ADE1 hmrΔ::ADE1 leu2-HOcs ade3::GAL-HO ade1 lys5 trp1::hisG ura3-52* | Pâques et al., 1998 |
| YLL3665 | YFP17 *RPB2-3HA::TRP1* | This study |
| tGI354 | *ho hml****∆****::ADE1 MATa-inc hmr****∆****::ADE1 ade1 leu2-3;112 lys5 trp1::hisG ura3-52 ade3::GAL::HO arg5,6::MATa::HPHMX* | Saponaro et al., 2010 |
| YLL3656 | tGI354 *rad51****∆****:: KANMX4* | This study |
| YLL3667 | tGI354 *RPB2-3HA::TRP1 rad51****∆****:: KANMX4* | This study |

**References**

Clerici M, Mantiero D, Lucchini G, Longhese MP. 2006. The *Saccharomyces cerevisiae* Sae2 protein negatively regulates DNA damage checkpoint signalling. *EMBO Rep* **7**:212-218. doi: 10.1038/sj.embor.7400593.

Clerici M, Trovesi C, Galbiati A, Lucchini G, Longhese MP. 2014. Mec1/ATR regulates the generation of single-stranded DNA that attenuates Tel1/ATM signaling at DNA ends. *EMBO J* **33**:198-216. doi: 10.1002/embj.201386041.

Lee SE, Moore JK, Holmes A, Umezu K, Kolodner RD, Haber JE. 1998. *Saccharomyces* Ku70, Mre11/Rad50 and RPA proteins regulate adaptation to G2/M arrest after DNA damage. *Cell* **94**:399-409. doi:10.1016/S0092-8674(00)81482-8.

Mantiero D, Clerici M, Lucchini G, Longhese MP. 2007. Dual role for *Saccharomyces cerevisiae* Tel1 in the checkpoint response to double-strand breaks. *EMBO Rep* **8**:380-387. doi:10.1038/sj.embor.7400911.

Pâques F, Leung WY, Haber JE. 1998. Expansions and contractions in a tandem repeat induced by double-strand break repair. *Mol Cell Biol* **18**:2045-54.

Saponaro M, Callahan D, Zheng X, Krejci L, Haber JE, Klein HL, Liberi G. 2010. Cdk1 targets Srs2 to complete synthesis-dependent strand annealing and to promote recombinational repair. *PLoS Genet* **6**: e1000858. doi: 10.1371/journal.pgen.1000858.
