# Supplementary material for: Resection is responsible for loss of transcription around a double-strand break in Saccharomyces cerevisiae
Source: eLife. 2015 Jul 31;4:e08942. doi: 10.7554/eLife.08942 (PMC4541074; doi:10.7554/eLife.08942)
Supplement: Supplementary file 2. — Primer sequences used in this study. DOI: http://dx.doi.org/10.7554/eLife.08942.014 [file elife08942s005.doc]

**Supplementary file 2.** Oligonucleotides used for qRT-PCR and ChIP

| **Name** | **Sequence** |
| --- | --- |
| *ACT1* fwd | 5’-AGAGTTGCCCCAGAAGAACACC-3’ |
| *ACT1* rev | 5’-CAAGGACAAAACGGCTTGGATGG-3’ |
| *ALG9* fwd | 5’-CCAGGTGATTTTCCAGAGAG-3’ |
| *ALG9* rev | 5’-GGCCACTCTTTACCGGTATC-3’ |
| *SNT1* fwd | 5’-CGAGCACCATAACACACACC-3’ |
| *SNT1* rev | 5’-TTGATAATGCCGAGGGAAAG-3’ |
| *ELO2* fwd | 5’-GTGGAAGGAATGGGTTAC-3’ |
| *ELO2* rev | 5’-GGGAAATACAAGTGAACTGC-3’ |
| *RRP43* fwd | 5’-GTTTTCCGTCTTGTATCCCG-3’ |
| *RRP43* rev | 5’-CAGTGCGACTCAAGACTAC-3’ |
| *PHO87* fwd | 5’-CACCTACATCTTCCAGCATG-3’ |
| *PHO87* rev | 5’-CATGAATCTACGTTCCCAGG-3’ |
| *BUD5* fwd | 5’-CTTCAGAGACTGGACAACTG-3’ |
| *BUD5* rev | 5’-GCCAATAAGACTCTACCCAG-3’ |
| *TAF2* fwd | 5’-ACGTCGTTGTTAATGGTGGTG-3’ |
| *TAF2* rev | 5’-CGCGAGTCTTATGCCAAAAA-3’ |
| *PER1* fwd | 5’-GAATCATACGCGAAGAGGGAG-3’ |
| *PER1* rev | 5’-CAACATTCCCGCCACAGTAAC-3’ |
| *IMG1* fwd | 5’-CGAGAGAATCGACTTGCTAACC-3’ |
| *IMG1* rev | 5’-CGACATCCAACCTTGTACCTCT-3’ |
| *BUD23* fwd | 5’-TTTCCCTCCCTTCTTCAGTGC-3’ |
| *BUD23* rev | 5’-CGATCCAATGGCTGTGCAATG-3’ |
| *ARE1* fwd | 5’-CTACCAGACCAAGGATAACG-3’ |
| *ARE1* rev | 5’-GGGTAGTTGATCTGGTACACG-3’ |
| *NFS1* fwd | 5’-CTACTTTCATACTGACGCCG-3’ |
| *NFS1* rev | 5’-CATAGATGGCACCTATTCCC-3’ |
| *DCC1* fwd | 5’-GACATTGAGGACGAGTTC-3’ |
| *DCC1* rev | 5’-CCACTGAGCTATAAACGG-3’ |
| *BUD3* fwd | 5’-TCTCCTACAATCCCTCTCAG-3’ |
| *BUD3* rev | 5’-GAAGACGCAGATACCTCTAC-3’ |
| *GBP2* fwd | 5’-GGCATGGAAGTAGAAGGTAG-3’ |
| *GBP2* rev | 5’-CTGGTATCTTCAAGGTCCTC-3’ |
| *SGF29* fwd | 5’-GAGAGATCCTGAACCTGATG-3’ |
| *SGF29* rev | 5’-CTTGCCAGAACCTTAGTACC-3’ |
| *VHR2* fwd | 5’-AAGCCAGCGTGAAGGAAGAA-3’ |
| *VHR2* rev | 5’-GTACAGGTGGAAGAGTGCGG-3’ |
| *ICL1* fwd | 5’-ACATCCCACAGAGAAGCCAAG-3’ |
| *ICL1* rev | 5’-TTGCGTCCCACCTCTGTAAC-3’ |
| *RRT13* fwd | 5’-GTGAGTGGATCTGAAGGTTTGC-3’ |
| *RRT13* rev | 5’-TTCTACCGCTGCCACAAGTG-3’ |
| *MOT2* fwd | 5’-GATTGCCATGCCCAAATCCC-3’ |
| *MOT2* rev | 5’-GTTCCGCCACTTTGCTGTTG-3’ |
| *RNR1* fwd | 5’-CTACGGCATGTGGGATTGGG-3’ |
| *RNR1* rev | 5’-TGGGATGTGGATGCAGTAGG-3’ |
| *VTC1* fwd | 5’-GTGTTCTTTGCCAATGAGCG-3’ |
| *VTC1* rev | 5’-TCCTGCACTGACCCTACCTA-3’ |
| *ALD5* fwd | 5’-GTCACTTTGGAGCTGGGAGG-3’ |
| *ALD5* rev | 5’-CGCAGCAAACTTCACCAGAG-3’ |
| *RPS24A* fwd | 5’-ACGTCTTGCACCCAAACAGA-3’ |
| *RPS24A* rev | 5’-AAACCGAAAACGGAGACAGC-3’ |
| *PTP3* fwd | 5’-GTTCTCCCTCTCCGCGTAAC-3’ |
| *PTP3* rev | 5’-GTTTGTTTGTGGTGCAGGCA-3’ |
